# Examining the Effects of Mental Health and Parent–Youth Relationship on the Associations Between Childhood Violence Exposure and Adolescent Dating Violence Perpetration †

**DOI:** 10.3390/children12050628

**Published:** 2025-05-13

**Authors:** Katie N. Russell, Laura A. Voith

**Affiliations:** 1 School of Social Work, University of Central Florida, Orlando, FL 32816, USA; 2 Mandel School of Applied Social Sciences, Case Western Reserve University, Cleveland, OH 44106, USA; lav41@case.edu

**Keywords:** adolescent dating violence, intimate partner violence exposure, child maltreatment, mental health, parent–child relationship

## Abstract

Background/objectives: Adolescent dating violence (ADV) is a serious public health concern affecting youth worldwide. Potential risk factors of ADV include child maltreatment (CM) and intimate partner violence exposure (eIPV), though existing research on ADV perpetration specifically is inconsistent. There is limited research on co-occurring eIPV and CM, despite co-occurrence in 30–60% of homes where there is one. This study aims to address these gaps by testing the impact of childhood violence exposure on ADV perpetration and assessing two potential, theory-informed mitigating factors—mental health and parent–youth relationship. Methods: This study utilizes moderated-mediation structural equation modeling with longitudinal data from a sample of 2354 U.S. adolescents (10–18) and one of their caregivers. Three models were tested: (1) childhood violence exposure (eIPV only, CM only, or co-occurring CM & EIPV) and ADV perpetration; (2) mediation by mental health quality on model 1; and (3) overall moderation by parent–youth relationship quality on model 2. Results: The final sample consisted of 961 youth with a history of dating. A significant direct effect between eIPV and ADV perpetration was found. There was a significant direct effect between co-occurrence (eIPV & CM) and ADV perpetration, which was mediated by mental health quality. Conclusions: This study further emphasizes the relation between eIPV and ADV and provides novel evidence of the impact co-occurrence has on ADV. Evidence of a trauma-informed mitigating factor, mental health quality, offers a potential point of intervention to be considered by mental health providers and ADV prevention/intervention programs.

## 1. Introduction

Adolescent dating violence (ADV), which is defined as threatening, stalking, and/or aggressive behavior (physical, sexual, and/or psychological) between adolescent dating partners (current or previous), impacts millions of adolescents worldwide each year [1]. A recent analysis of the 2018 World Health Organization (WHO) ADV data found that the global prevalence rate for physical and sexual ADV in the past year was approximately 16%, with great variation across countries [1]. In the same study, the prevalence rate in the United States (U.S.) specifically was greater than 20% [1]. With the inclusion of psychological and cyber ADV, a systematic review by Tomaszewska & Schuster found that prevalence rates ranged from 25% to nearly 96% across North America and Europe [2]. Despite the detriment of ADV alone, the harm does not stop there. Research has linked ADV with countless pernicious outcomes, including, but not limited to, depression symptoms, suicidal ideation, bullying, eating disorders, and community violence perpetration [3,4].

### 1.1. Child Maltreatment and Adolescent Dating Violence

Child maltreatment (CM), broadly defined as neglect, threatening harm, physical, emotional, or sexual abuse, and/or trafficking of a minor, is another major public health concern, with prevalence rates as high as 75% in some countries [5]. In the U.S. specifically, it is estimated that one in seven children falls victim to child maltreatment each year [6]. A small, existing body of literature reports a positive association between CM and ADV, though measurement differences exist between studies, limiting cross-study comparison. Within this developing area, few studies separate ADV victimization and perpetration, choosing instead to use an overall ADV composite score [7]. Karsberg et al. assessed ADV victimization and perpetration separately and found CM to significantly predict both [8]. To advance this nascent field, it would be valuable to separate ADV victimization and perpetration experiences until the etiological nuances between them, as they relate to CM, can be determined. Parenting practices, empathy in relationships, and attachment style mediate the relation between CM and “overall” ADV [9,10]; however, studies must disentangle these relationships as they relate to victimization or perpetration to aid the field with more targeted prevention and intervention. Further advancing the field, risk and protective factors grounded in theory will strengthen the field’s understanding of explanatory mechanisms, aiding prevention/intervention efforts and future research.

### 1.2. Childhood IPV Exposure and Adolescent Dating Violence

Childhood exposure to intimate partner violence (eIPV), which includes witnessing (hearing or seeing) or dealing with the aftermath of physical, psychological, sexual, or threatening violence between caregivers, impacts around 25% of children worldwide [11]. In the U.S., an estimated 15.5 million children experience eIPV each year, equating to approximately 20% [12]. The body of literature examining eIPV as an antecedent of ADV is much more robust than the CM and ADV fields. Studies examining eIPV and ADV also have more depth, with far more studies examining ADV victimization and perpetration separately. Studies report a consistent, positive association between eIPV and ADV victimization; however, findings related to ADV perpetration are less consistent [3,13]. Two factors likely contributing to the inconsistency across studies are measurement (e.g., ADV type, timeframe) and sample (e.g., high-risk groups vs. public, setting, age) differences [3]. Many studies have examined underlying protective and risk factors shaping the relation between eIPV and ADV, including attitude towards dating violence, gender, anger regulation, and prosocial peers [13,14,15,16,17]. However, few studies explicitly utilize theory to guide factor selection or explain these relationships. Theoretically supported studies examining underlying protective and risk factors to better understand the relation between eIPV and ADV perpetration are essential to advance the field. Also, notably, Coulter & Mercado-Crespo found that in homes where eIPV is occurring, there is as high as a 60% likelihood of co-occurring CM [18]. Despite this finding, only one study, to the author’s knowledge, has since examined the impact of co-occurring eIPV and CM on ADV [19]. Additional research should examine this co-occurrence, as well as potential factors specific to the nuances of its relationship with ADV.

### 1.3. Theoretical Support

Potentially traumatic events are distressing occurrences that can harm an individual’s body or mind, including eIPV and CM [20]. Trauma responses are characterized by overwhelming an individual’s capacity to cope, from which a heightened sense of physiological arousal occurs [21]. If one’s physiological arousal remains heightened, changes in the body and brain may occur [22] and have lasting effects on mental health, including persistent agitation, anxiety, and depression. Mental health challenges further inhibit an individual’s ability to cope with trauma and future challenges [23]. Unresolved trauma from childhood exposures such as IPV or maltreatment can manifest in multiple facets of one’s life, including behavioral and emotional triggers, increased aggression, and negative relationship outlooks [22]. It is also suggested that an inability to cope can lead individuals to be re-exposed to situations “reminiscent of the original trauma” [24]. This re-enactment of trauma can perpetuate further traumatic victimization or perpetration of violent acts against others [24].

Despite the potential effects of trauma, many who experience eIPV or CM grow up to have healthy relationships. Given this multi-finality, it is clear that children’s trajectories are also dependent on their distinctive developmental and socio-ecological context, as supported by the developmental risk and resilience model [3,25]. This model suggests that unhealthy outcomes are not solely due to behaviors and choices made, but also risk factors out of one’s control (e.g., demographics, adversity) [26]. However, the model posits that not all individuals are destined for negative outcomes solely because of risk factors, either, as protective factors (e.g., parenting, empathy) also play a major role in outcome multi-finality [26].

Combining the developmental risk and resilience model and trauma theory can advance a strong conceptual model to examine ADV as an outcome of eIPV or CM. Some pertinent factors derived from this combined model include parenting and youth mental health [3].

### 1.4. Current Study

Inconsistent results across studies examining ADV perpetration as an outcome of eIPV and the limited literature examining the impact of CM and co-occurrence (eIPV and CM) on ADV perpetration warrant additional research. This study aims to add to the field using a large, nationally representative dataset that assesses all forms of ADV perpetration. Additionally, this study seeks to address the theoretical gap in examining risk and protective factors across all three fields by testing a combined model stemming from trauma theory and the developmental risk and resilience model. Specifically, this study will examine the mediating effect of mental health quality (trauma theory) and the moderating effect of parent–youth relationship quality (developmental risk and resilience) on the relationship between childhood violence exposure (CM & eIPV) and ADV perpetration. The research questions (RQs) for this study are as follows:

- RQ1: does eIPV, CM, or co-occurrence of both impact the likelihood of ADV perpetration among youth?
  ○ Hypothesis: endorsement of eIPV, CM, or co-occurrence will increase the likelihood of ADV perpetration among youth.
- RQ2: does youth mental health quality mediate the relation between childhood violence exposure (i.e., eIPV, CM, co-occurrence) and ADV perpetration?
  ○ Hypothesis: mental health quality will mediate the relation between childhood violence exposure and ADV perpetration among youth, with better mental health quality decreasing the likelihood of ADV perpetration when eIPV, CM, or co-occurrence are endorsed.
- RQ3: how does parent–youth relationship quality moderate the mediating effect of mental health quality on the relationship between childhood violence exposure (i.e., eIPV, CM, co-occurrence) and ADV perpetration among youth?
  ○ Hypothesis: parent–youth relationship quality will moderate the mediating effect of mental health quality on the relationship between childhood violence exposure and ADV perpetration among youth, with better parent–youth relationship quality decreasing the likelihood of ADV perpetration in addition to the impact of mental health quality.

## 2. Materials and Methods

This study used data from the National Survey of Teen Relationships and Intimate Violence (STRiV) [27]. The initial goal of the STRiV study was to assess the prevalence rates of ADV in a nationally representative sample of youth, with all forms of ADV included, as well as individual and interpersonal risk factors of ADV. This study builds on the initial goal of the STRiV by not only considering risk factors of ADV but also potential protective factors mitigating that risk. This article is an extended version of our paper presented at the 2025 Society for Social Work Research Conference [28].

### 2.1. Data Collection

The STRiV recruited youth (10–18) and one of their caregivers from a nationally representative sample of 5105 U.S. households from 2013 to 2020. A web-based survey was used at six annual waves, with data from 2645 caregivers and 2354 youth from primarily urban households (86.3%) for the wave 1 survey. This study utilizes wave 1–4 data, with youth retention at 62.5% for wave 2, 69.7% for wave 3, and 69.9% for wave 4. Waves 5 and 6 were not included, given that a large portion of the sample was over the age of 18 at those timepoints. Caregivers reported a median household income of $67,500, 70.0% owned their residences, and 68.6% completed at least some college. On average, youth were 14 years of age at enrollment, with a nearly equal number of boys (50.0%) and girls (50.0%). Most youth and their caregivers reported being White, non-Hispanic (62.5%) or Hispanic (22.0%). Additional demographic information can be found in Table 1 and Table 2.

### 2.2. Measures

The waves from which each variable was derived were as follows: wave 1; eIPV and CM, wave 2: mental health and parent–youth relationship quality; and waves 3–4: ADV perpetration.

### 2.3. Adolescent Dating Violence Perpetration

The Conflict in Adolescent Dating Relationships Inventory (CADRI) was used to measure ADV [29]. Of the 62 items, 31 measured ADV perpetration (i.e., psychological, threatening, sexual, physical, and relational) and were used to address the aims of this study. Youth were also only asked to complete the CADRI if they reported a prior history of dating at that wave. The CADRI asks youth to report behaviors they have exhibited in dating relationships in the past year using a 4-point Likert scale ranging from 0 = Never to 3 = Often [29]. To include the largest possible sample of youth while maintaining temporal order, this study utilized data from waves 3 and 4. For youth with a reported history of dating at both waves, the higher of the two scores was used. A continuous composite score, ranging from zero to 93, was created for each wave, with higher values representing higher rates of ADV perpetration. The CADRI had a Cronbach’s alpha of 0.92 for this study, indicating excellent reliability.

### 2.4. Childhood Violence Exposure

eIPV. Two dichotomous (yes/no), youth-report items modeling Straus et al. and Felitti et al. were used to measure eIPV [30,31]. Youth were asked about lifetime exposure to physical IPV in the home between a caregiver and their partner. One item assessed seeing a caregiver get slapped, punched, pushed, hit, or beaten up, and the other assessed hearing any of those things. The data were operationalized with a binary flag variable equaling 1 if “yes” was selected for either item, indicating a history of eIPV, and 0 if “no” was selected for both items. These items had a Cronbach’s alpha of 0.84 for this study, indicating good reliability.

CM. Caregivers were asked four questions regarding the frequency at which they physically harmed (“pushed, grabbed, slapped, or hit”), threatened physical harm, criticized, and yelled or shouted at their child in the last month. Caregivers responded on a 5-point Likert scale ranging from 1 = “Never” to 5 = “Very Often”. For this study, each item was re-coded to reflect endorsement of CM = 1 or no endorsement = 0. Using Gluck’s definition of emotional maltreatment, caregiver responses of 4 = “Often” or 5 = “Very Often” were re-coded to = 1 (CM) for the two items assessing “yelling and shouting” and “criticizing”. For the items assessing “threatening harm” and “physically harming” the child, any response greater than 1 = “never” was re-coded = 1 (CM), and “never” was re-coded as 0 [32]. The responses for the four items were summed and then dichotomized, with any values greater than zero representing CM. These items had a Cronbach’s alpha of 0.60 for this study, indicating acceptable reliability.

Overall Childhood Violence Exposure (Co-Occurrence). Using the variables above, four mutually exclusive categories (eIPV only, CM only, co-occurrence, none) were created to represent overall childhood violence exposure. The if-then function was utilized to code the variable as follows: (a) if neither form of childhood violence exposure was endorsed = 0 (none); (b) if only eIPV was endorsed = 1 (eIPV only); (c) if only CM was endorsed = 2 (CM only); and (d) if both were endorsed = 3 (co-occurrence). For the analysis, this variable was then used to create three dummy variables for each type of violence exposure (eIPV only, CM only, co-occurrence), with “none” representing the reference group.

### 2.5. Mental Health Quality

Mental health quality was measured using the 5-item Mental Health Inventory (MHI) [33]. Youth were asked to respond to each item on a 6-point Likert scale, ranging from 1 = All the time to 6 = None of the time. For the current study, two items required reverse coding (e.g., how much of the time, during the past month, have you felt calm and peaceful?). All five items were then summed for a total score, ranging from five to 30, with higher scores indicating better mental health quality [33]. For this study, the Cronbach’s alpha was 0.80, indicating good reliability.

### 2.6. Parent–Youth Relationship Quality

To measure parent–youth relationship quality, the STRiV modeled eight items from the National Longitudinal Study of Adolescent to Adult Health questionnaire [34]. Youth were asked to indicate whether they agree or disagree with each of the statements pertaining to their relationship with their caregiver on a 5-point Likert scale ranging from 1 = Strongly Agree to 5 = Strongly Disagree. For the current study, responses for each of the items were reverse-coded and summed, resulting in higher scores indicating positive parent–youth relationship quality. Mirroring previous research, the summed score was divided by eight to calculate an average parent–youth relationship quality score (ranging from one to five) and then re-coded to create a categorical variable with three categories: negative quality (score ≤ 2.5), average quality (score = 2.6–3.9), and positive quality (score ≥ 4) [35]. This scale had a Cronbach’s alpha of 0.87 for this study, indicating good reliability.

### 2.7. Data Analysis Plan

Prior to testing the proposed model, descriptive statistics were run to better understand the sample and missingness, as well as to check the assumptions of SEM. First, univariate analyses (e.g., mean, standard deviation [SD], skewness, kurtosis) were conducted with demographic variables and each of the variables in the model to test for normality and preliminary missing data, as well as to understand the sample. Then, bivariate analyses, including inter-variable correlations and the deviation from linearity test, were conducted to evaluate the variables for collinearity and linearity. Finally, variable inflation factor (VIF), Pearson’s r, and tolerance analyses were conducted to test all variables for multicollinearity.

This study utilized the AMOS package to test the overall goodness of fit (*df*, CFI, Chi square, TLI, *p*-value, and RMSEA) for the conceptual model using structural equation modeling (SEM) [36]. For models with zero degrees of freedom, IFI was calculated to assess goodness of fit in place of TLI [37]. To answer each of the RQs for this study, three models were tested: (1) RQ1: modeling the relation between childhood violence exposure, using the categorical co-occurrence variable, and ADV; (2) RQ2: modeling mediation by mental health quality on the first model, with nonsignificant pathways from model 1 removed; and (3) RQ3: a moderated mediation model testing overall moderation by parent–youth relationship quality on the second model. SEM was selected to test the overall model given its ability to assess the overall goodness of fit for complex models. In particular, SEM provides an advantage of simultaneously testing each pathway in the final model, rather than separating each pathway using other analysis strategies, as well as producing regression weights for the individual paths in post-hoc analysis [38]. Though minimal for the final sample (<1%), missing data were handled using full information maximum likelihood (FIML) method to calculate parameter estimates [39]. Direct and indirect effects were interpreted using standardized regression weights for models 1 and 2 and unstandardized regression weights for model 3.

## 3. Results

### 3.1. Descriptive Statistics

For childhood violence exposure, 65.0% reported none (*n* = 1719), 20.3% reported child maltreatment only (*n* = 538), 9.4% reported IPV exposure only (*n* = 249), and 5.3% reported co-occurrence of child maltreatment and IPV exposure (*n* = 139). A total of 961 youth reported a history of dating and, therefore, were asked about their own ADV perpetration. This information can also be found in Table 1. A total of 61.4% of youth endorsed ADV perpetration of some type, with a mean score of 3.69 (*SD* = 6.10), and the most frequently reported type of ADV was verbal/emotional (66.5%). Mental health quality scores ranged from 6 to 30, with a mean score of 23.08 (*SD* = 4.14). The majority of youth reported positive parent–youth relationship quality (65.9%), 33.5% reported average quality, and 6.6% reported negative quality.

### 3.2. Model 1 (RQ1): Childhood Violence Exposure & ADV

For research question 1, it was hypothesized that eIPV, CM, and the co-occurrence of both would all significantly increase the likelihood of ADV perpetration among youth when compared with youth not exposed to either. In partial support, model 1 indicated a statistically significant direct pathway between eIPV and ADV (*β* = 0.13, *p* < 0.001) and between co-occurrence and ADV (*β* = 0.12, *p* < 0.001). There was no significant pathway between CM and ADV. Therefore, it was removed prior to testing model 2. Overall goodness of fit for this model was demonstrated with a chi-square value of 0.00 (CFI = 1.00, IFI = 1.00 [*df* = 0], RMSEA = 0.125). Though the RMSEA value is high, the CFI value indicates a good fit. However, given the large sample size and relatively small effect size, the results specific to the relation between co-occurrence and ADV should be interpreted with caution. Further details can be found in Table 3.

### 3.3. Model 2 (RQ2): Mediation Model

For research question 2, it was hypothesized that youth mental health quality would mediate the relation between all forms of childhood violence exposure and ADV perpetration. This hypothesis was partially supported, as the direct effect between co-occurrence and ADV became nonsignificant, and the pathways from co-occurrence to mental health quality (*β* = −0.24, *p* = 0.002) and mental health quality to ADV (*β* = −0.21, *p* < 0.001) were significant with a modest effect size. This suggests that for youth who experienced co-occurring eIPV and CM, ADV scores were significantly higher when mental health quality was poorer. However, the direct effect of eIPV on ADV remained significant (*β* = 0.14, *p* = 0.032), with no significant pathway from eIPV to mental health quality. This indicates that no mediation was found in the relation between eIPV and ADV. Given that the pathway in model one between child maltreatment and ADV was nonsignificant, child maltreatment was not included in this model, and therefore, mediation was not tested on the pathway. Overall model goodness of fit for this model was demonstrated with a chi-square value of 0.00 (CFI = 1.00, IFI = 1.00 [*df* = 0], RMSEA = 0.40). Further details can be found in Table 4.

### 3.4. Model 3 (RQ3): Moderated Mediation Model

For research question 3, it was hypothesized that parent–youth relationship quality would significantly moderate the overall mediational model (model 2) between all forms of childhood violence exposure, mental health quality, and ADV perpetration. To test this, multiple-group analysis was conducted in AMOS on the mediation model using the three parent–youth relationship quality groups: positive, average, and negative quality. The hypothesis was not supported by the model, as demonstrated by a nonsignificant *p*-value, as well as minimal change between the unconstrained model and the equal structural weights model (∆CFI = 0.002, ∆*x*^2^ = 6.87). The chi-square value for the equal structural weights model was 79.29 (CFI = 0.940, TLI = 0.905, RMSEA = 0.085), indicating overall goodness of fit for the model. With a small sample size for the negative quality group (*n* = 21), the model was also tested with collapsed groups (positive and average/negative). However, results did not significantly differ between the two models, and therefore, the initial grouping structure was maintained. Further details can be found in Table 5.

## 4. Discussion

The findings from this study are additive to the field in several ways. Not only does this study add to the existing body of literature by providing additional evidence of the link between eIPV and ADV perpetration, but it also shows novel evidence of the link between co-occurrence and ADV perpetration. The findings highlight nuances specific to the co-occurrence of eIPV and CM and illuminate a theoretically driven factor that may be a potential point of intervention with ADV perpetration among youth exposed to this co-occurrence: mental health quality. All of these findings have several implications for research and practice in this field moving forward.

### 4.1. Childhood Violence Exposure and ADV

In the present study, the rates of reported childhood violence exposure and subsequent ADV perpetration were all slightly higher than or nearing the high end of rates previously reported in the literature. Over one-third of youth reported at least one of the two forms of childhood violence exposure studied (CM and eIPV). Approximately one in six youth were reported to have experienced CM, a slightly higher prevalence than previously noted in the literature for U.S. youth (i.e., one in seven) [6]. More than 25% of youth reported eIPV, aligning closely with global prevalence rates and slightly higher than previously reported for the U.S. specifically [11,12]. Just over 60% of youth endorsed some form of ADV perpetration, falling toward the higher end of prevalence rates reported in the literature for U.S. youth [1,2]. Given that this study utilizes the largest nationally representative dataset measuring all forms of ADV perpetration in U.S. youth, with others only measuring a few specific types of ADV (e.g., only physical and sexual) or all forms with smaller samples, this study emphasizes the importance of including all types of ADV when reporting prevalence rates [3].

This study’s findings specific to the relation between childhood violence exposure and ADV perpetration are also additive to the field. First, the statistically significant relation between eIPV and ADV perpetration provides the currently discordant field with additional evidence of the association using the largest nationally representative dataset with all forms of ADV perpetration. Second, despite the results being nonsignificant for the relation between CM alone and ADV perpetration, the association between co-occurrence and ADV perpetration was significant, highlighting the nuance in the two forms of violence exposure co-occurring, which the literature suggests befalls 30–60 percent of households where eIPV is present [40]. These findings also align with the combined trauma theory and developmental risk and resilience model proposed, in that youth exposed to these traumatic events were more likely to perpetrate violence “reminiscent of the original trauma”, but not all youth that experienced those same forms of trauma did so [24,25]. This also emphasizes the need for providers and ADV program leaders to take a trauma-informed, person-centered approach in helping youth heal from childhood violence exposure.

### 4.2. Mental Health Quality

In addition to the direct effects identified in this study, the findings also indicated that mental health quality mediated the pathway between co-occurrence and ADV. Though factors representing mental health have previously been considered in the literature exploring the relationship between eIPV and ADV and CM and ADV, most studies did not include a theoretical framework guiding model building, limiting study rigor, nor did any study consider the effect of mental health solely on ADV perpetration [10,14,41,42]. Furthermore, this is the first study to consider mental health as it relates to the complex relation between the co-occurrence of these two forms of violence and ADV perpetration. This finding aligns with the principles of trauma theory, suggesting that being exposed to violence in the home impacts youth mental health quality, thus impeding youth from being able to cope with future issues and re-exposing them to circumstances reminiscent of the initial trauma (i.e., ADV) [24]. However, aligning with the developmental risk and resilience model, this finding also provides a potential point of intervention for youth exposed to co-occurring eIPV and CM and at risk of perpetrating ADV, which is imperative to the field of ADV prevention and intervention programming [43]. This finding also further emphasizes the need for trauma-informed, person-centered approaches to the prevention of ADV, given the harm that can come from childhood violence exposure and the impact that untreated mental health outcomes can have on the likelihood of youth becoming perpetrators of violence themselves [22].

### 4.3. Parent–Youth Relationship Quality

Though this study did not identify moderation by parent–youth relationship quality, there are still implications stemming from this finding. First, it is possible that moderation was not identified due to a very small sample of youth reporting negative parent–youth relationship quality (*n* = 21). Also, given that parenting practices and attachment style have been found to have an impact on eIPV and ADV, and CM and ADV, respectively, it is possible that specific facets of parent–youth relationship quality have more of an impact than overall parent–youth relationship quality [9,19,44]. Further investigation should consider individual aspects of parent–youth relationships, such as parenting practices, trust, or closeness, in addition to overall parent–youth relationship quality, in the relation between childhood violence exposure and ADV. In doing so, it would align with the postulation of the developmental risk and resilience model that youth relationships, particularly with their family, can serve as a protective factor decreasing the likelihood of harmful developmental outcomes such as ADV perpetration [26]. This study was also the first, to the author’s knowledge, to consider parent–youth relationship specific to the nuanced relation between co-occurrence of both forms of violence and ADV perpetration, so additional investigation into these relations may still be warranted.

### 4.4. Limitations

This study is not without its limitations. First, only data regarding physical eIPV were collected for the STRiV dataset at the included waves. Though the relation between physical eIPV and ADV perpetration was significant and the endorsement rate for eIPV was higher than previously reported in the literature the prevalence and strength of the relation could be underestimated without measuring other forms of eIPV (e.g., psychological, threatening) [12]. In collecting information on only one form of eIPV, nuances specific to how the different forms of eIPV (i.e., physical, sexual, psychological) inform the differing types of ADV perpetrated could also not be explored. For instance, it is possible that exposure to one form of eIPV may then relate more strongly to the perpetration of that same form of ADV (e.g., psychological eIPV and ADV). Further, the protective factors that mitigate the relation between different eIPV and ADV perpetration may differ by type of violence, which would align with the trauma theory notion of re-enacting violence reflective of the violence experienced [24]. There were also some limitations regarding the CM measurement methods. First, there is a potential for response bias given that caregivers were asked to report on their own perpetration of CM, and youth reports were not included to either corroborate or illuminate their interpretation of the experience. Furthermore, despite the CM prevalence rates in this study being higher than those reported in the literature, neglect was not included in the measurement of CM, therefore failing to capture one of the more prevalent forms of CM and possibly producing underestimates of CM occurrence in this sample [5,6]. This measurement limitation could have contributed to the nonsignificant effect of CM on ADV perpetration for this study, particularly given that previous research has found a link between neglect and subsequent violence perpetration in adolescence [45]. However, in combination with another form of trauma (i.e., IPV exposure), the other forms of CM that were measured in this study were still found to be significant.

Regarding the measurement of parent–youth relationship quality, as noted previously, it is possible that particular components of parent–youth relationships are more impactful on the relation between childhood violence exposure and ADV, such as parenting practices or attachment [9,44]. Also, using a smaller assessment tool, which touched on broader aspects of the parent–youth relationship, as well as a categorical variable to assess its effect on the relation between childhood violence exposure and ADV, may have limited the specificity of the variable and possibly reduced the potential for a significant effect. Finally, given that youth gender was collected from caregivers using a binary option and information specific to sexual orientation and gender-diverse identities was not collected from youth until later waves in the STRiV study, our ability to understand the nuances in the significant effects identified in this study as they relate to youth identity was limited.

### 4.5. Practice Implications

With nearly 40% of youth in this study reporting some form of childhood violence exposure and, subsequently, 61% of youth with dating histories reporting ADV perpetration, the findings of this study further illuminate the urgency of intervening in the cycle of violence and suggest that prevalence may be even higher than previously believed. Given the direct effect that childhood violence exposure has on ADV perpetration, practitioners (e.g., school counselors, child therapists) should consider conducting screenings for childhood violence exposure to identify at-risk youth for ADV prevention efforts. Furthermore, though several ADV prevention and intervention programs exist and continue to be developed, previous research has encouraged the use of theoretically driven mitigating factors in programming to minimize inconsistency in program effectiveness [43]. This study offers one such potential point of intervention informed by theory for ADV programs to consider—mental health quality. Given its mediating effect, ADV programs should consider incorporating a mental health module that takes early exposure to potentially traumatic events, such as childhood violence exposure, into account. For instance, programs could coach youth on an array of coping skills with opportunities for practice, as well as provide examples of what a healthy relationship should look like, given that youth could be exhibiting behaviors they have been exposed to at home [24]. In doing so, programs may help to mitigate the harmful effects of trauma on youth’s ability to cope with future stressors, such as those that can occur in adolescent relationships, and provide them with a working model of what is or is not a healthy behavior in a relationship. Additionally, given the multi-finality of ADV perpetration for youth exposed to violence, providers should consider applying a trauma-informed, person-centered approach in helping youth heal from childhood violence exposure to prevent ADV perpetration. Providers and ADV prevention programs should avoid tactics that seek to blame youth for not knowing what a healthy relationship looks like, given the likelihood that nearly half of them could be victims themselves, and should instead work to identify additional points of intervention that are effective for youth from different identities and with varying childhood experiences.

### 4.6. Research Implications

The results of this study also have several implications for future research. First, despite the high prevalence rates of childhood violence exposure found in this study, future research should include all forms of eIPV and CM to provide a more complete picture of how widespread these issues are. This would also allow researchers to consider how different types of violence exposure relate to the different types of ADV perpetration. Additionally, future research should consider including more detailed information about eIPV and CM, such as frequency and severity, to make further distinctions regarding how they impact the likelihood of youth perpetrating ADV and to add depth and nuance to the current understanding of these relations. Second, research should continue investigating potential mitigating factors in the relation between eIPV and ADV perpetration that are informed by theory, whether applying the same theories or others (e.g., social learning theory, ecological systems). Researchers should also consider the relation between childhood violence exposure and ADV perpetration, as well as the potential mitigating factors in that relation, from an intersectional lens and including youth perspectives. This could be done by using community-based participatory action methods with diverse youth to parse how the relations differ for varying identities of youth, as well as to identify protective and risk factors specific to varying identities. As suggested by the developmental risk and resilience model, the lives of youth do not occur in a vacuum; lived experiences impact the multi-finality of their experiences and behaviors. It stands to reason, then, that the research should also consider the lived experiences of those being studied and how that might impact what will or will not serve as a point of intervention to prevent ADV perpetration for those youth. Finally, though this study focused on identifying nuances specific to the co-occurrence of eIPV and CM, future researchers should also consider other forms of violence and their relation to ADV perpetration, such as other types of peer violence (e.g., sibling violence, bullying), community violence, and systemic violence.

## 5. Conclusions

Overall, this study provides additional evidence of the relation between eIPV and ADV perpetration, as well as novel evidence of the nuanced impact of the co-occurrence of both forms of violence exposure on ADV perpetration. Additionally, this study offers youth mental health quality as a theoretically informed point of intervention for ADV programming to consider. 

## Figures and Tables

**Table 1:** Youth Characteristics (N = 2354).

Characteristics	*n* (%)	*M (SD)*
Age		13.79 (2.60)
Gender		
Boy	1176 (50.0)	
Girl	1178 (50.0)	
Race		
White, non-Hispanic	1471 (62.5)	
Black, non-Hispanic	219 (9.3)	
Other, non-Hispanic	80 (3.4)	
2+ Races	66 (2.8)	
Hispanic	518 (22.0)	
Violence Exposure		
Childhood IPV exposure only	538 (22.8)	
Child maltreatment only	249 (10.6)	
Co-occurrence	139 (5.9)	
None	1428 (60.7)	
History of dating (*n* = 1646)	961 (58.4)	
ADV perpetration reported	590 (61.4)	
Sexual Orientation at Wave 4 (*n*= 1499)		
Heterosexual	1378 (83.7)	
Gay or Lesbian	20 (1.2)	
Bisexual	52 (3.2)	
Other	14 (0.9)	
Don’t know/Not sure	32 (1.9)	
Refused	3 (0.2)	

**Table 2:** Caregiver Characteristics (N = 2354).

Characteristics	*n* (%)	*M (SD)*
Gender		
Man	1176 (50.0)	
Woman	1178 (50.0)	
Marital status		
Married	1793 (76.2)	
Widowed	12 (0.5)	
Separated	52 (2.2)	
Divorced	206 (8.8)	
Never married	129 (5.5)	
Not married, living with partner	174 (7.4)	
# of Marriages		2.16 (0.67)
Household size		4.25 (1.36)
Education level completed (*n* = 1646)		
Less than high school	117 (7.1)	
Some high school or diploma/GED	400 (24.3)	
Some college	502 (30.5)	
Bachelor’s degree or higher	627 (38.1)	
Employment type		
Employed	1438 (61.1)	
Self-employed	222 (9.4)	
Unemployed	635 (27.1)	
Retired	34 (1.4)	
Household ownership status		
Owned	1648 (70.0)	
Rented	662 (28.1)	
Other	44 (1.9)	
Household income		
Less than $10,000	148 (6.3)	
$10,000–$20,000	162 (6.9)	
$20,001–$30,000	225 (9.6)	
$30,001–$40,000	254 (10.8)	
$40,001–$50,000	194 (8.2)	
$50,001–$60,000	202 (8.6)	
$60,001 or higher	1169 (49.7)	

**Table 3:** Model 1—Childhood Violence Exposure and ADV Perpetration (N = 961).

Pathway	Direct Effect			
	β	B	S.E.	*p*-Value
IPV exposure → ADV perpetration	0.13	2.48	0.62	<0.001
Co-occurrence → ADV perpetration	0.12	2.82	0.76	<0.001
Child maltreatment → ADV perpetration	0.02	0.28	0.50	n.s.

Model fit: *x*^2^ = 0.00; *df* = 0; CFI = 1.00; IFI = 1.00; RMSEA = 0.125. Note. n.s. = not significant.

**Table 4:** Mediation Effect of Mental Health Quality on Childhood Violence Exposure & ADV—Standardized Weights (N = 961).

Pathway	Direct Effect	*p*-Value	Indirect Effect	Mediation Conclusion
IPV exposure → MHQ	0.09	n.s.	-	-
Co-occurrence → MHQ	−0.24	0.002	-	-
MHQ → ADV perpetration	−0.21	<0.001	-	-
IPV exposure → MHQ → ADV perpetration	0.14	0.032	−0.02	Not identified
Co-occurrence → MHQ → ADV perpetration	−0.01	n.s.	0.05	Identified

Model fit: *x*^2^ = 0.00; *df* = 0; CFI = 1.00; IFI = 1.00; RMSEA = 0.40. Note. n.s. = not significant; MHQ = Mental health quality.

**Table 5:** Moderated Mediation Model with Parent-Youth Relationship Quality as Moderator (N = 961).

Model	*x* ^2^	*df*	∆ *x*^2^	∆ *df*	*p*-Value	CFI	∆ CFI	Moderated Mediation Conclusion
Unconstrained	72.42	14	-	-	<0.001	0.942	-	Not identified
Equal Structural Weights	79.29	19	6.87	5	n.s.	0.940	0.002	

Model fit: TLI = 0.905; RMSEA = 0.085. Note. n.s. = not significant.

## Data Availability

Data is available at https://www.icpsr.umich.edu/web/NACJD/studies/36499 (accessed on 7 May 2025).
